# Editorial: Pharmacological interaction between drugs and medicinal plants

**DOI:** 10.3389/fphar.2022.1081090

**Published:** 2022-12-14

**Authors:** Myrna Déciga-Campos, Rosa Ventura-Martínez, Maria Eva González-Trujano, Dâmaris Silveira

**Affiliations:** ^1^ Sección de Estudios de Posgrado e Investigación, Escuela Superior de Medicina, Instituto Politécnico Nacional, Ciudad de México, México; ^2^ Departamento de Farmacología, Facultad de Medicina, Universidad Nacional Autónoma de México, Ciudad de México, México; ^3^ Laboratory of Neuropharmacology of Natural Products, National Institute of Psychiatry Ramón de la Fuente Muñiz, Mexico City, Mexico; ^4^ Laboratory of Natural Products, Faculty of Health Sciences, University of Brasilia, Brasilia, Brazil

**Keywords:** drugs combination, interactions, synergism, traditional medicine (TM), medicinal plants/evaluation

The use of medicinal plants as complementary and alternative medicine (CAM) has been widely increasing worldwide (Welz et al., 2018). According to the WHO (World Health Organization), between 10 and 50% of the population in developed countries use herbal products on a regular basis (WHO, 2021). The global herbal medicines market moved USD 151.91 billion in 2021 and is projected to be worth USD 347.50 billion by 2029, growing at a CAGR of 11.16% (GLOBENEWSWIRE, 2022). Its commercialization has increased in part due to the pharmaceutical industry offering products for human consumption that contain medicinal plants and/or their metabolites in pharmaceutical forms such as tablets, capsules, or syrups (GLOBENEWSWIRE, 2022). The market reflects the importance of medicinal plants or their metabolites in therapeutic protocols, stimulating research on the efficacy and safety of herbal medicines.

The use of medicinal plants or their metabolites has not only significantly increased worldwide: research by scientific groups has also expanded, demonstrating their effectiveness in treating diseases with minimal or no adverse effects. People are convinced that natural products are less harmful than synthetic drugs because they are of natural origin, i.e., “if they are not good, they are not bad either.” However, herbal medications and supplements contain chemical compounds whose interactions might produce the same benefits or risks as those of other pharmacologically active compounds, whether natural or synthetic (Asher et al., 2017). Therefore individuals, mainly patients exposed to polypharmacy regimens, should be aware of drug-drug, herbal-herbal, herbal-food, or herbal-drug interactions.

In this way, researchers have increased their efforts to demonstrate both the efficacy of medicinal plants and/or their metabolites for the treatment of various diseases and their safety, either on their own or in combination with other drugs.

Preclinical studies have grown in recent decades to demonstrate that interactions between conventional drugs and extracts of medicinal plants and/or their metabolites can be considered a good therapeutic strategy, according to the research about combined therapies. The purpose of those interactions is to improve treatments’ efficacy while reducing potential adverse effects. Nevertheless, clinical studies evaluating these interactions are still limited or inconclusive. However, although such benefits can become a reality in several interactions, negative results might also be possible. Therefore, research should be encouraged to reveal the frequency and nature of these interactions.

As previously mentioned, extracts of medicinal plants contain more than one possible bioactive metabolite responsible for therapeutic activity. In this respect, administering herbal medicine alone or combined with conventional drugs can modify the intensity of an active compound’s effects depending on its chemical nature and how it works. This could influence the outcome of treatment in at least three cases: additive, supra-additive (synergistic), or infra-additive (antagonistic) effects (Tallarida, 2012).

Although there has been an increase in the number of papers on the subject, little is still known about herb-drug or herb-herb interactions, in part, because i) pharmacovigilance is weak regarding herbal medicine use; ii) adverse/unexpected effects are usually not notified, since patients do not tell their doctor they are using herbal medicine in combination with other drugs; or iii) most of the available papers on the subject refer to *in vitro* results, which are a useful tool for warning about potential deleterious effects, but the *in vivo* experiments that support them are minimal for some plants or still missing for others that are already therapeutically used in traditional or folk medicine.

Therefore, this Frontiers Research Topic, “Pharmacological Interaction Between Drugs and Medicinal Plants,” contributes, as a collection of eight articles written by 62 authors (total views: 17,351 on 29 November 2022). Four of these publications are original research articles and four are reviews, and they describe or integrate preclinical scientific knowledge focusing on medicinal plants and drug interactions.

First, an interesting review describes the strong antioxidant activity of propolis, which could strengthen the therapeutic effect of some drugs such as antimicrobials, hyperglycemics, and those used to improve cognition and movement. In the case of chemotherapy, propolis improved not only antitumoral activity but also attenuated multiorgan toxicity (Hossain et al.). The second review refers to the evidence available in electronic databases and focuses on the combined use of natural products and tamoxifen in breast cancer, identifying the current gaps and suggesting possible future studies for improving treatment strategies. Additionally, this review considers information on some metabolites that can interact with tamoxifen, improving its pharmacokinetic parameters (Yen et al.).

The third review presents interesting information about probiotics combined with Chinese herbal medicines (CHM) for treating ulcerative colitis. Probiotics are bioactive microorganisms that positively impact the body’s health when consumed in adequate doses and combined with CHM. This therapy generates a therapeutic synergism by inhibiting the abnormal inflammatory response, protecting the intestinal barrier, and restoring the intestinal microbiota imbalance (Hu et al.). The fourth and final review in this collection shows that the use of warfarin with some traditional Chinese medicine (TCM) products plays an important role in herb interactions. Warfarin has a long pharmacokinetic half-life in a therapeutic window, and interactions with drugs, herbs, or food can cause severe adverse events. The complex composition of CHM may cause dual effects such as potentiating and attenuating the anticoagulant effect of warfarin, so patients should exercise caution when using warfarin and herbal remedies concurrently (Zhuang et al.).

These four literature reviews emphasize the importance of pharmacokinetic and pharmacodynamic studies in the interactions of clinical drugs with medicinal plants.

Regarding pharmacological interactions of medicinal plants, including some pharmacokinetic phases, there is currently not much information on the metabolic and transporter targets due to the complexity of medicine based on medicinal plant extracts. An original study in the present collection analyzes, *in vitro*, the pharmacokinetic ADME (absorption, distribution, metabolism, and excretion) system in preparations based on traditional Latin American and European herbs usually integrated into the Brazilian Unified Health System (SUS). Continuous research on these herbal preparations is important because it allows the mapping of potential ADME effects in different populations (Dias Araujo Mazzari et al.).

The second manuscript provides evidence about the importance of studies to identify the kind of drug-herb interactions that occur during some pharmacokinetic phases. *Salix cortex* extracts, at high concentrations, interact with CYP3A4, one of the CYP450 isoforms most used by many drugs for their hepatic metabolism. The authors showed the importance of considering that due to polypharmacy, people are at risk of inducing adverse effects when they use more concentrated or long-term herbal preparations that can interact with some cytochromes, leading to changes in their metabolism (Dutra Gomes et al.). Due to the potential interaction with many herbs, it is important to investigate the inherent population at risk and the long-term use of *Salix cortex* extracts.

Regarding the effects of positive drug interactions, it has been shown that the concomitant use of *Coriolus versicolor* products and drugs against cancer, such as tamoxifen, can improve their therapeutic effect. The extract stabilizes the biochemical changes of long-term therapy with tamoxifen, benefiting patients with compromised immune systems. The results also showed that *C. versicolor* products do not inhibit tamoxifen metabolism *in vitro*; however, clinical studies to confirm preclinical results will always be needed (Razmovski-Naumovski et al.). The last original research of this collection describes the hepatoprotective effects of a polysaccharide obtained from *Smilax china* L, a well-known medicinal plant with a variety of pharmacological properties. This study emphasized the importance of protection against acetaminophen-induced hepatotoxicity produced by N-acetyl-L-cysteine (NAC), an approved treatment (Wang et al.).

There is little information available globally about how frequently medicinal plants are used as a form of treatment in patients with a variety of conditions or whether the plants are used as the only treatment or as a complement to the drugs prescribed by clinicians. In many cases, medicinal plants are used as CAM and as traditional and local medicine in aqueous preparations such as infusions (tea) or decoctions. For example, 67.4% of patients with generalized anxiety disorders used medicinal plants, as recorded in a public hospital in Mexico (IMSS). However, in this study, the authors did not report whether patients used the medicinal plants to complement their conventional treatment or alone (Romero-Cerecero et al., 2019).

Since there is a lack of information about scientific studies on interactions between drugs and medicinal plants around the world, this collection, which includes eight manuscripts on the topic “Pharmacological Interaction Between Drugs and Medicinal Plants,” represents an example of the importance of gathering evidence about the pharmacological interactions between drugs for clinical use and medicinal plants. This will help to identify their potential in producing maximum efficacy with fewer adverse effects, contributing to an optimal alternative in the treatment of several conditions. Ongoing work on the subject is necessary to ensure that herbal medicines are both effective and safe, encouraging rational use of this therapeutic option.
